# Experimental Insights into Concrete Flow-Regimes Subject to Shear-Induced Particle Migration (SIPM) during Pumping

**DOI:** 10.3390/ma13051233

**Published:** 2020-03-09

**Authors:** Shirin Fataei, Egor Secrieru, Viktor Mechtcherine

**Affiliations:** Institute of Construction Materials, Technische Universität Dresden, 01187 Dresden, Germany; shirin.fataei@tu-dresden.de (S.F.); egor.secrieru@tu-dresden.de (E.S.)

**Keywords:** fresh concrete, rheology, lubricating layer, polydispersed granular phase, aggregate volume-fraction

## Abstract

In this paper, the authors have focused on shear-induced particle migration (SIPM), its effect on concrete flow patterns, and lubricating layer formation during pumping. For this purpose, various volume-fractions *ϕ* of aggregates were selected. The particle migration was analyzed by applying two methods: sampling hardened concrete exposed to pumping and performing X-ray microcomputed tomography (μCT) and image analysis to determine the thickness of the lubricating layer due to SIPM. The results indicate that the first approach is unsuitable due to the nearly equal molecular density of particles and matrix. The second approach indicated that the actual thickness of the lubricating layer depends on the discharge rate as well as on *ϕ* and viscosity of concrete bulk; hence, it cannot be defined as a constant parameter for all concrete mixtures. Additionally, the concrete pipe-flow pattern, i.e., plug versus shear flow, was captured and studied while considering pumping pressure and discharge rate. It was concluded that particle migration is essential in the cases of both flowable and very flowable concretes with a high volume-fraction of solids. The changes in rheological properties caused by SIPM are severe enough to influence the definition of the flow pattern as plug or shear and the discharge rate of pumped concrete as well.

## 1. Introduction

In concrete rheology, the granular nature of concrete is often neglected when performing rheological investigations or studying flow behavior with respect to various applications. This being so, it is generally assumed that the concrete’s constituents are uniformly distributed throughout the sampling volume during rheological measurements with wide-gap rheometers [1,2]. Another prominent example is pumping, during which the concrete flow in the pipeline is expected to reach a steady state after an initial critical deformation [3]. From this point on, concrete is composed of two immiscible fluids: the lubricating layer and concrete bulk [4,5]. It is assumed that each of these fluids deforms according to its rheological properties. The rheological properties of the lubricating layer are considered similar to that of constitutive mortar; however, its thickness is still in debate, with 2 mm being the most accepted value [6,7,8].

Concrete as a viscous granular suspension is a manifestation of the migration of solid inclusions in an inhomogeneous flow field occurring during pumping. According to [3], the flow-induced particle migration (FIPM) is a generic term combining the effects of shear, gravity, and wall geometry. Shear-induced particle migration (SIPM) and the wall-effect are the most determining phenomena behind the formation of the lubricating layer; indeed, the effect of gravity can be disregarded during pumping due to the dynamic character of the material flow. In the paper at hand, the main focus lies on SIPM. During pumping, the particles tend to migrate away from the pipe walls, where the highest shear rates develop toward the centerline, resulting in a non-uniform distribution of solid particles [9,10]. While this phenomenon is not critical in conventional concretes with a high yield stress, its influence on the rheological properties and pumping behavior of high-performance concretes is pronounced [3,11]. High-performance concretes have low water-to-binder ratios and contain a high dosage of chemical admixtures to enhance the workability of fresh concrete. As well, they have a lower amount of water and a correspondingly larger fraction of solid particles to enhance mechanical performance [7]. As a result the mixtures are densely packed and their rheological properties are influenced even by slight changes in the volume-fraction of the granular phase [12,13,14].

Phillips et al. [9] proposed a constitutive equation for computing the particle concentration and velocity fields in the case of dense, mono-dispersed granular suspensions based on the work of Leighton and Acrivos [15]. Shauly et al. [16] expanded this model to deal with polydispersed concentrated suspensions with discrete and continuous particle size distributions. However, these models contain phenomenological constants that can only be obtained by experimental fitting of the particle distribution. In considering SIPM in concrete pipe-flow formulation, the main challenges are the complexity of concrete composition containing a high volume-fraction of polydispersed particles whose size ranges between several tens of nm and several mm and the lack of quantitative experimental assessment due to concrete’s opacity. Therefore, the determination of particle distribution in concrete during pumping is not possible with nuclear magnetic resonance imaging as performed in [9,16].

To investigate the influence of SIPM on coarse particles of concrete during pumping, the authors focused on particles larger than 1 mm, assuming that aggregates of an irregular shape with continuous size distribution are suspended in a fine homogenous, cementitious mortar. This paper treats the experimental analysis of the SIPM of aggregates larger than 1 mm and its influence on the pumping behavior of conventional vibrated concrete (CVC) and self-compacting concrete (SCC). New approaches for capturing the thickness of the lubricating layer and the non-uniform particle distribution resulting from SIPM during pumping are introduced. According to the results the current methodologies which generally consider concrete as two homogeneous fluids—concrete bulk and lubricating layer—with defined rheological properties determined using rheometers are insufficient for predicting the pumping behavior of modern concretes [17,18,19]. By studying the concrete-flow regime, the authors concluded that the influence of SIPM on rheological properties of mixtures with a high volume-fraction of solid particles is significant during pumping. To derive a reliable correlation between pumping pressure, discharge rate, and the rheological properties of concrete, SIPM must be incorporated into the pipe-flow equations.

## 2. Theoretical Background

### 2.1. Rheology of Concrete—Single-Phase Fluid or Multiphase Suspension?

The rate of deformation for any fluid undergoing shear stress depends on its apparent viscosity η, which is a constant for Newtonian fluids or a function of further parameters in the case of non-Newtonian fluids. The relation between shear stress and shear rate for concrete can be written as [20]:

(1) $$\tau =\eta \left(\left|\dot{\gamma }\right|,\;\phi ,T,\;t\ldots \right)\;\dot{\gamma }$$

where $\dot{\gamma }$ and $\left|\dot{\gamma }\right|$ are the shear rate (1/s) and the magnitude of strain rate tensor (1/s), respectively. ϕ, T, and t correspond to the volume-fraction of solid particles in a suspension, the temperature, and the time after the addition of water. The fresh concrete can be approximated as a single-phase fluid, meaning a homogenous fluid with uniform rheological properties throughout the whole volume of the sample. The simple rheological models, such as the Bingham model, only consider the shear rate as an influential parameter in the viscosity function; cf. (2) and (3):

(2) $$\tau \geq \tau_{0}:\;\tau =\left(\mu +\;\frac{\tau_{0}}{\left|\dot{\gamma }\right|}\right)\;\dot{\gamma }$$

(3) $$\tau <\tau_{0}:\;\dot{\gamma }=0$$

with $\tau_{0}$ and μ the rheological parameters yield stress (Pa) and plastic viscosity (Pa·s). These parameters can be experimentally determined using rheometers. Usually, the experiments are performed at a defined time to eliminate the time-dependency of the mixtures. Moreover, they are considered constant for a given mixture and independent of the possible changes in the distribution of the granular phase.

Alternatively, concrete can be considered as a multiphase suspension, which apparent viscosity depends not only on the intensity of shear rate, but also on the volume-fraction ϕ of solids. Studies indicate that yield stress and plastic viscosity of concrete are functions of the reduced volume-fraction of particles $\phi /\phi_{c}$, where $\phi_{c}$ is the maximum dense packing fraction of solid particles. This relation is linear for dilute suspensions (ϕ<0.05) and exponential for semi-dilute suspensions ($\phi /\phi_{c}<0.78$). According to [12,14] the general formulations for Bingham parameters of a suspension, $\tau_{0}\left(\phi \right)$ and μ(ϕ), considering the volume-fraction ϕ and the maximum packing fraction of particles $\phi_{c}$ can be written as:

(4) $$\tau_{0}\left(\phi \right)=\tau_{0}\left(0\right)\sqrt{\frac{1-\phi }{{\left(1-\frac{\phi }{\phi_{c}}\right)}^{m_{\tau }}}}$$

(5) $$\mu \left(\phi \right)=\mu \left(0\right){\left(1-\frac{\phi }{\phi_{c}}\right)}^{-m_{\mu }}$$

where $m_{\tau }$ and $m_{\mu }$ are the material constants and $\tau_{0}\left(0\right)$ and μ(0) are the yield stress and plastic viscosity of the fluid at zero particle concentration.$\;\phi_{c}$ can be determined experimentally using the approach of de Larrard [21]. These material constants are obtained by fitting the outcomes of the rheological measurements. Thus, the influence of the granular phase on the rheological properties of concrete, e.g., during pumping and 3D printing, can be accounted using multiphase rheological models such as Equations (4) and (5).

### 2.2. Pumping of Concrete—Modeling as Two Immiscible Single-Phase Fluids or MultiPhase Suspension with Varying Rheological Properties?

The single-phase approach for the analysis of concrete pumping behavior assumes that the pumped concrete consists of two immiscible fluids described by Equations (2) and (3). One fluid is a thin layer of fine mortar that is formed in the vicinity of the wall [4]. This so-called lubricating layer (LL) does not exceed a few mm and exhibits distinctly different rheological properties from the concrete bulk, which is the second fluid under consideration. This fluid either slides over the lubricating layer as a solid plug or is fully/partially sheared, where large shear deformations are concentrated in the lubricating layer [5,22]. In terms of the Bingham model, the lubricating layer has a much lower yield stress and plastic viscosity than does the concrete bulk.

Considering the rheological properties of both concrete bulk and the lubricating layer, the relationship between pumping pressure loss ΔP (Pa), and the discharge rate Q (m^3^/s) in a pipeline of length L (m) and radius of $R_{Pipe}$ (m) can be represented according to Equation (6) [23]:

(6) $$\begin{array}{c} Q=\int_{R_{LL}}^{R_{Pipe}}2\pi rU_{LL}dr+\int_{R_{Plug}}^{R_{LL}}2\pi rU_{SB}dr+\int_{0}^{R_{Plug}}2\pi rU_{PB}dr\\ =\frac{\pi }{24\mu_{LL}\mu_{B}}[3\mu_{B}\Delta P/L\left(R_{Pipe}^{4}-R_{LL}^{4}\right)-8\tau_{0,LL}\mu_{B}\left(R_{Pipe}^{3}-R_{LL}^{3}\right)\\ +3\mu_{LL}\Delta P/L\left(R_{LL}^{4}-R_{Plug}^{4}\right)-8\tau_{0,B}\mu_{LL}\left(R_{LL}^{3}-R_{Plug}^{3}\right)] \end{array}$$

where $U_{LL}$ (m/s), $U_{SB}$ (m/s), and $U_{PB}$ (m/s) are the velocity in the lubricating layer, shearing zone and plug zone of concrete bulk, respectively. $R_{LL}=R_{Pipe}-e_{LL}$ (m) and $R_{Plug}=2\tau_{0,B}L/\Delta P$ (m) are respectively the lubricating layer and plug radii. $\tau_{0,LL}$ (Pa), $\mu_{LL}$ (Pa·s), $\tau_{0,B}$ (Pa), and $\mu_{B}$ (Pa·s) are the yield stress and plastic viscosity of the lubricating layer and bulk. $e_{LL}$ (m) is the thickness of the lubricating layer [23]. The ΔP−Q relationship is approximated under two major assumptions: (1) the lubricating layer and concrete bulk are homogeneous single-phase fluids with unchanging Bingham parameters, and (2) the thickness of the lubricating layer $e_{LL}$ is constant. It is reported to be between 1 and 8 mm based on experimental investigations [6,7,24].

The multiphase approach is more complex in comparison to its single-phase version. Here, the impact of the granular phase and interparticle interactions on the properties of concrete bulk and the lubricating layer during pumping must be considered and implemented in the flow formulations. The shear-induced particle migration (SIPM) and the wall-effect are addressed as the main mechanisms behind the formation of the lubricating layer. The rheological properties of concrete and the lubricating layer as well as the thickness of the lubricating layer are investigated with regards to SIPM. Consequently, the derived pumping formulation is linked to changes in the granular phase. To do so, deeper insights and more experimental evidence on shear-induced particle migration of concrete during pumping are required.

**Figure 1:**
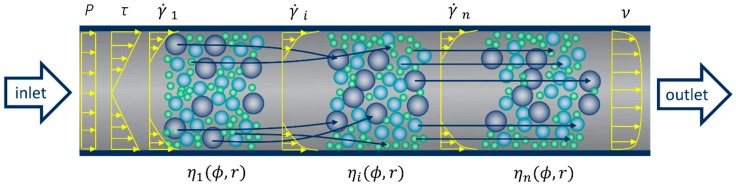
Particle migration during pumping before reaching a steady state.

Shear-induced particle migration occurs when the granular phase of concrete is subject to non-uniformly distributed shear stresses and shear rates; see Figure 1 for the pumping process.

Due to the non-uniform distribution of shear stresses over the cross-section, forces of different magnitudes are acting on the various particle surfaces. The resultant forces induce a radial displacement of the particle in addition to its transient displacement along the pipeline [25]. The larger particles migrate toward the center of the pipeline with lower shear rates, and the water and finer particles move to the wall to fill the space; see arrows in Figure 1. The non-uniform particles distribution induces different local viscosities in the radial direction ($\eta_{i}\left(\phi ,r\right)$). The increase in local viscosities from the wall to the centerline hinders the migration and eventually results in equilibrium and steady state ($\eta_{n}\left(\phi ,r\right)$) after a critical deformation [3].

The suspension balance model [10] and diffusive flux model [9,26] are the most commonly used approaches with regard to shear-induced particle migration. The former assumes that particle migration occurs due to variations in normal stress, while the latter targets the differences in shear rate as the main reason. The suspension balance model is considered to be a more generalized approach; however, the diffusive flux model is often preferred due to its simplicity and relatively high accuracy [26,27,28].

Based on the diffusive flux model, Phillips et al. [9] developed a constitutive equation for a Newtonian concentrated suspension that derives the time-dependent and steady state local particle concentration and velocity profiles. They showed that the conservation equation of solid particles composes of motions due to spatially varying interaction frequency and spatially varying viscosity; see Equation (7):

(7) $$\frac{\partial \phi }{\partial t}+\frac{\partial (\upsilon_{z}\phi )}{\partial z}=\frac{a^{2}}{r}\frac{\partial }{\partial r}\left\{rK_{c}\phi \dot{\gamma }\left(\frac{\partial \phi }{\partial r}+\frac{\phi }{\dot{\gamma }}\frac{\partial \dot{\gamma }}{\partial r}\right)+rK_{\eta }\left(\dot{\gamma }\phi^{2}\right)\frac{1}{\eta }\;\frac{\partial \eta }{\partial \phi }\frac{\partial \phi }{\partial r}\right\}$$

where a is the particle radius (m), $K_{c}$ and $K_{\eta }$ are dimensionless phenomenological constants obtained by fitting the equation to experimentally observed local particle distributions. Shauly et al. [16] adapted Equation (7) for polydispersed systems with discrete and continuous particle distributions. Validation of such models for concrete requires the correct determination of particle distribution, which is no straightforward procedure in opaque concrete.

Spangenberg et al. [3] showed that in the case of pumping, shear-induced particle migration (SIPM) and the wall-effect are more dominant than gravity-induced particle migration. Each can cause 3–5% variation in the local volume-fraction of aggregates when compared to the initial volume-fraction of the mixture. In a pipeline, the wall-effect occurs instantaneously, and for SIPM Spangenberg et al. estimated a characteristic time $T_{c}^{FIPM}$ for reaching the steady state as:

(8) $$T_{c}^{FIPM}=\frac{R_{Pipe}^{2}}{10a^{2}\phi^{2}\dot{\gamma }}$$

where $\dot{\gamma }=V/R_{Pipe}$ and V is the average velocity (m/s). It follows that larger particles have a shorter characteristic time, which means that they reach steady state in shorter time and length. Since the finer particles might still migrate in the radial direction, the rheological properties of the material are influenced as a result.

## 3. Materials and Methods

### 3.1. Materials

Two types of concrete were considered: conventional vibrated concrete (CVC) and self-compacting concrete (SCC). The mixture compositions are given in Table 1, which specifically details compositions of the constitutive mortars and aggregates used. The constitutive mortar with a maximum aggregate size of one mm and the aggregates larger than one mm will be referred to as “mortar” and “aggregates”, respectively. This arrangement was set based on the definition of the lubricating layer in this work, the thin layer formed in the vicinity of the pipe wall during pumping consisting of a fine cementitious mortar with a maximum particle size of 1 mm.

The name of each mixture is composed of a capital letter indicating whether the concrete is of type CVC or SCC and a number giving the volume-fraction of aggregates larger than 1 mm in size. For example, C52 is a CVC mixture with aggregates larger than 1 mm taking 52% of its volume. The additional “c” in C47c and S42c marks the replacement of round 8–16 mm size aggregates with crushed basalt aggregates of 8–11 mm and 11–16 mm. Since according to Equation (7) the volume-fraction of aggregates is one of the most decisive parameters in evaluating SIPM [3,9], it was varied by ± 5% volume-fraction in the case of the mixtures containing round aggregates. Thus, three different aggregates’ contents were investigated for both CVC and SCC. Naturally, when the volume-fraction of aggregates increases by 5%, the volume-fraction of mortar decreases by 5%, and vice versa. To reduce the deviation between the properties of the constitutive mortars, the proportions among all components were kept constant. The authors are aware of possible changes in the rheological properties of concrete and constitutive mortar due to higher or lower content of fine aggregates of 0–1 mm and corresponding variations in specific surface area. This aspect is considered below in the discussion of the outcomes.

Mixing and testing were performed in the laboratory at a temperature of 26.6 ± 2 °C and a relative humidity of 49% ± 7%. The water and aggregates were cooled prior to mixing to achieve a concrete temperature of 20 ± 2.5 °C. The maximum packing density of the aggregates was determined by de Larrard’s approach [21] and resulted in $\phi_{c}=0.719$ and $\phi_{c}=0.705$ for round and crushed aggregate combinations, respectively. In the next step, the reduced volume-fraction of aggregates, defined as $\phi /\phi_{c}$ [12], was calculated for each mixture; see Table 1.

### 3.2. Rheological Measurements

Rheological measurements were carried out on concrete and constitute mortar within an age span of 30–40 min. The fine mortar was obtained by wet-screening of the concrete on a 1-mm sieve [7,32].

**Figure 2:**
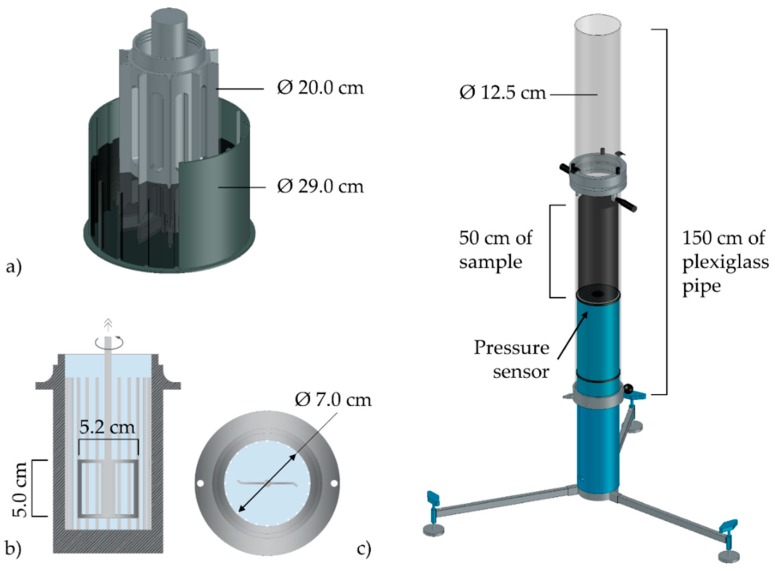
(**a**) ConTec 5 viscometer, (**b**) HAAKE MARS II rheometer, and (**c**) Sliding pipe rheometer (Sliper).

Table 2 and Figure 2 present an overview of the main testing devices. A coaxial wide gap concentric cylinder rheometer, and a rheometer equipped with a building material cell [33] were utilized to measure the rheological properties of concrete and mortar, respectively. Both geometries have ribs on outer cylinders to avoid slippage; see Figure 2a,b. During the test, the resisting torque *T* of the material is measured while applying a rate-controlled hysteresis loop with predefined rotational velocity *N*. The maximum rotational velocity was chosen to attain an approximated shear rate of 10 s^−1^ in the sample. For data evaluation, the methods listed in Table 2 were used. *R_o_*, *R_i_,* and *H* correspond to outer and inner radii, and the height of the sampling volume, respectively.

For SCC mixtures with shear-thickening behavior, the modified Bingham (MB) model was used for calculating the rheological parameters [34]. For CVC mixtures the formation of a plug region during the test was examined, and the yield stress and plastic viscosity values were corrected accordingly [35]. It is worth mentioning that applied rheometric devices have different measurement setups with different torque resolutions, which approximately correspond to 5–10 Pa and 4–5·10^−7^ Pa shear stress. Obviously, differences in the values of yield stress and plastic viscosity should be considered only if measured above the resolution range. Table 2 provides torque resolutions for both devices.

Kasten [36] developed an in-situ device called a sliding pipe rheometer (Sliper), which provides reliable estimation on the pumping pressure necessary for a desired discharge rate; cf. Figure 2c. The outcomes also provide information about the pressure loss in pipelines independently of the pipe length. The reliability of the measurements has been verified in two full-scale setups for various types of concrete [7,36]. The 1.5 m long sliding pipe is filled to one third with concrete and is moved in a controlled manner in the form of strokes. By installing different weights on the handles various velocities and, hence, different corresponding discharge rates can be generated while the pressure in concrete is measured by a sensor. The shear rate in the Sliper device can be adjusted according to the flow rate in the real pipeline, e.g., 5100 m^3^/h for CVC and 540 m^3^/h for SCC mixtures [32]. Indeed, the Sliper device can mimic the pressure loss per unit length of pipe, concrete flow behavior, including the hydrodynamics and particle–particle interaction, very well, and in addition predict the necessary pumping pressure for a given flow rate [37]. More information on data analysis can be found in [7,36].

### 3.3. Methodologies for Capturing the Lubricating Layer and Shear-Induced Particle Migration

The lubricating layer is usually defined either as the region with highest velocity gradient or as the region consisting of a fine cementitious mortar at the interface between concrete in relative motion and the pipe wall. In this work the lubricating layer and its thickness are defined as the region with very fine mortar containing aggregates smaller than 1 mm. To capture the real thickness of the lubricating layer, a combined approach was implemented: concrete sampling in the Sliper device and X-ray microcomputed tomography of the interface transition zone. Both methods are described below.

#### 3.3.1. Quantification of Radial/Angular Displacement of Aggregates in Fresh Concrete

Direct visual observation is a natural approach when studying particle motion. The shear-induced particle migration was reproduced by means of experiments with the Sliper at various velocities. During measurement in the sliding pipe, the deformation of the top surface of concrete was recorded to track the flow pattern for various discharge rates, i.e., for plug flow, partial shear, and full shear of concrete bulk. The initial evaluation of the videos indicated that with partial and full shear flows, the particles at the surface move/rotate toward the pipe wall. While inside the sample each particle is restricted from all sides, the particles positioned at the surface can move more freely. Still, the deformation on the free surface could represent deformations within the sample: On the free surface, the displacements of the particles are greater and the plug radius is smaller than inside the sample.

Theoretically, such radial and angular displacements can be traced back to the parabolic velocity profile of the sample during pumping, which consists of a non-zero resultant velocity vector on one side of the particle. The cross product of the particle lever arm by the resultant velocity vector renders the angular velocity. The superimposition of this angular displacement on the main longitudinal displacement results in the radial displacement of the particle. The radial and angular displacements increase with an increase in the size of particles. At the same time, the displacement of a particle in a densely packed suspension is limited by the rheological properties of its surroundings. According to Hagen-Poiseuille law the velocity magnitude over radius correlates with ΔP/η, which means the velocity decreases with increases in viscosity.

All things considered, the authors assumed that the radial velocity of the particles at the top is related to the degree and intensity of the curvature in the velocity profile generated under specific pressure loss ΔP. The evaluation of the videos offered the possibility of tracking few aggregates with a size range of 8–16 mm. Furthermore, the authors observed unexpected formation of plug flow in S47. This encouraged the authors to determine the experimental plug radii, if any, using video frames, and to compare them with the analytical plug radii obtained according to the expression $R_{Plug}=2\tau_{0,B}/\Delta P$, see for e.g., [38].

**Figure 3:**
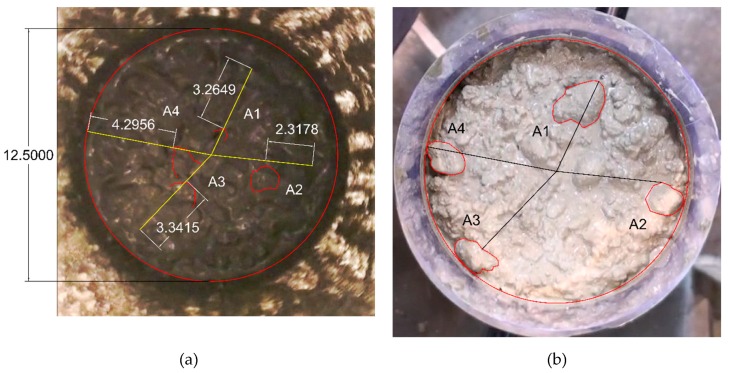
Displacement of four coarse aggregates in C47 between two frames no. (**a**) #90 and (**b**) #110; vertical velocity in the Sliper test was 0.72 m/s.

To determine the radial velocity of particles, a single stroke was chosen with a discharge rate of approximately $30{\;m}^{3}/h$ for CVC and $12.5{\;m}^{3}/h$ for SCC mixtures. These values refer to the maximum discharge rate in which few particles can still be detected and tracked. It is important to have similar discharge rates when comparing the radial velocity of the particles in each type of concrete. The video sequence was 25 frames per second. The visible coarse aggregates were traced in the subsequent frames; see Figure 3. The displacement of the particle was determined using the diameter of the pipe as the scale, i.e., 125 mm. The average radial velocity was calculated using the total displacement within two marked frames. In the initial frames, only the tips of some particles could be detected.

It is noteworthy that the radial displacement of each aggregate particle is influenced by its size, mass, and shape. The irregular shapes of the particles result in rotational motion in addition to the radial displacement. One of the reasons for assuming spherical particles in granular science is to avoid complications such as this.

#### 3.3.2. Sampling of Pumped Concrete

**Figure 4:**
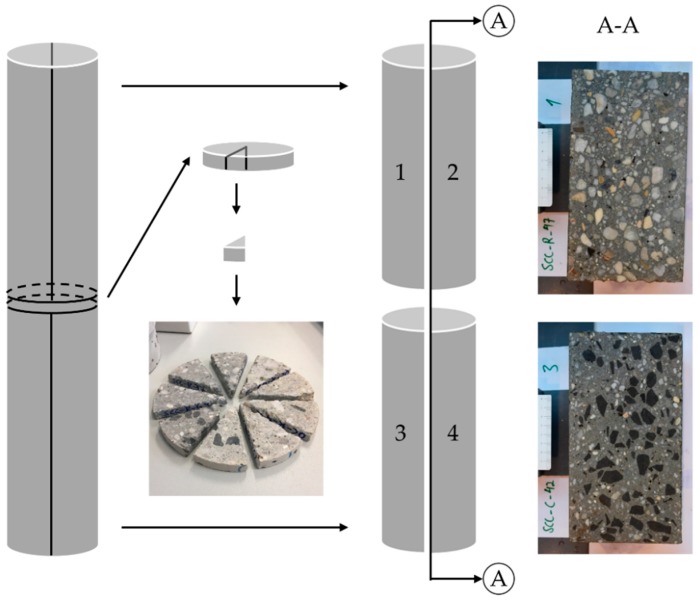
The cutting pattern for a “pumped” concrete specimen after hardening.

Prior to each measurement with Sliper, a tube with D=125 mm, made of a flexible transparent polymer sheet of 0.2 mm thickness, was fixed temporarily inside the upper pipe. This tube acted as an intermediary between the upper pipe and concrete. By the end of the Sliper measurements, the upper pipe, containing the “pumped” concrete sample was removed from the setup and stored for setting and hardening. This procedure was handled with care to prevent any deformation of the fresh sample. At an age of four hours, the hardened samples were removed from their confinement and stored according to DIN EN 12390-2 [39] at relative humidity of 100% and 20 °C room temperature for seven days and in the storage room with standard climate (20 °C/65% RH) for another 21 days. Finally, the samples were cut and prepared for two different tests. Figure 4 illustrates geometrical details of the prepared Sliper cylindrical specimens.

Thin discs were cut from the cylinders. They were purposely divided into nine circular sectors for X-ray microcomputed tomography (μCT). The two remaining cylinders were longitudinally cut along the A–A axis. The resulting half-cylinders (1 + 2 and 3 + 4) were visually assessed in respect of their lubricating layer thicknesses. The examples of the A–A cross-section in Figure 4 clearly indicate a lower concentration of coarse aggregates at the vicinity of the pipe wall due to the high shear rates in this region.

**Figure 5:**
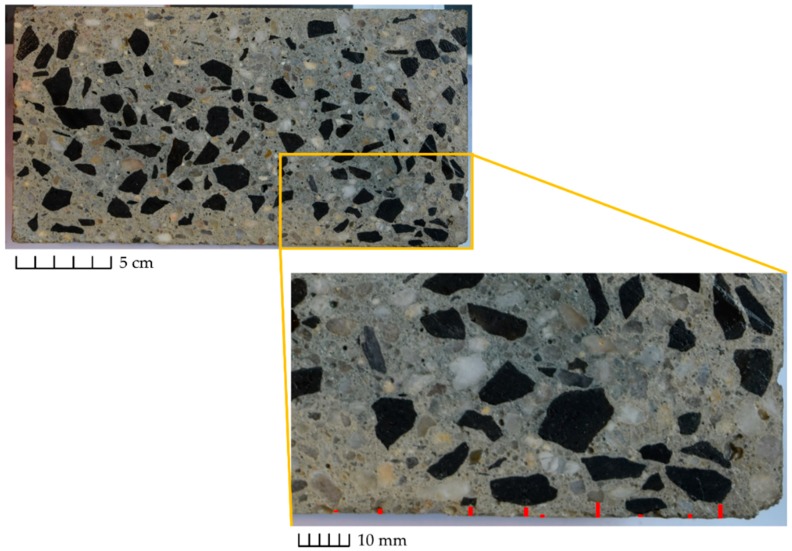
Determination of the lubricating layer thickness for C47c.

The contrast observed between aggregates and the matrix during the visual assessment of the half-cylinders offered the possibility to determine the lubricating layer thickness over the whole length of the Sliper samples. To analyze the images the software ImageJ (version 1.52m) was used. The maximum and minimum diameters were calculated to distinguish between different aggregate fractions. A short ruler later used as the scale for determining length was captured in all images. The distances between aggregates larger than 1 mm and the pipe wall, as marked in Figure 5 with red lines, were approximated along with the sample for the visible particles near the wall periphery. The average provided the experimental thickness of the lubricating layer for each mixture.

**Figure 6:**
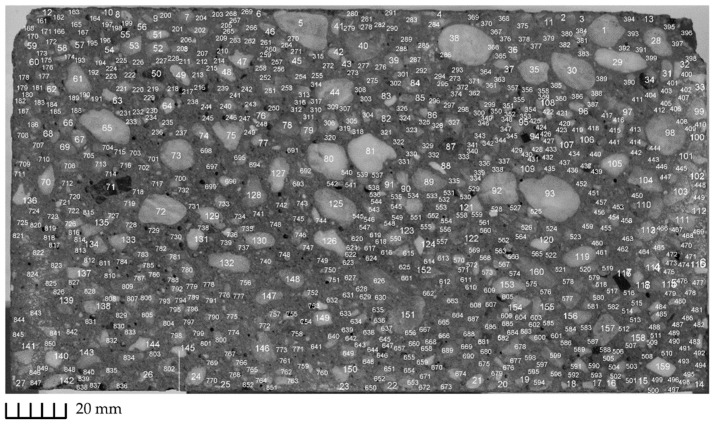
Visible particles larger than one mm for determination of the particle distribution for S47.

The visible particles larger than 1 mm were marked and the distance between the center of each particle to the wall was determined; see Figure 6. In a further step, the particles were categorized in four groups according to the size ranges, i.e., 1–2, 2–4, 4–8, and 8–16 mm. The objective was to identify the particle size distribution over the pipe cross-section corresponding to the specific size ranges. For this purpose, the particle surface area was quantified. It is worth mentioning that this approach is accompanied by a degree of inaccuracy: e.g., the aggregates of 8–16 mm might be labeled to be smaller than their actual size; the particles corresponding to size range 2–4 or 4–8 mm might indeed represent the “tip” of a larger aggregate, e.g., 4–8 mm or 8–16, respectively. This would result in a lower surface area for a particle size fraction 8–16 mm. In the case of C47c and S42c, the aggregates larger than 8 mm were of basalt type; see Figure 5 for C47c. Due to the black color of larger aggregates it was easy to distinguish between particles within the size range 1–8 mm and 8–16 mm. In an ongoing study by the authors, the employment of aggregates of distinct color should provide a more accurate determination of particle size distribution. Note that the determination of particle distribution is essential for deriving the dimensionless phenomenological constants, $K_{c}$ and $K_{\eta }$, specified in Equation (7). Currently the suggested values presented in [9,26] hold for mono-dispersed Newtonian suspensions. in the case of concrete within a pumping process, it is a dense polydispersed non-Newtonian suspension undergoing high shear rates inside a pipeline.

#### 3.3.3. X-ray Microcomputed Tomography (μCT)

**Figure 7:**
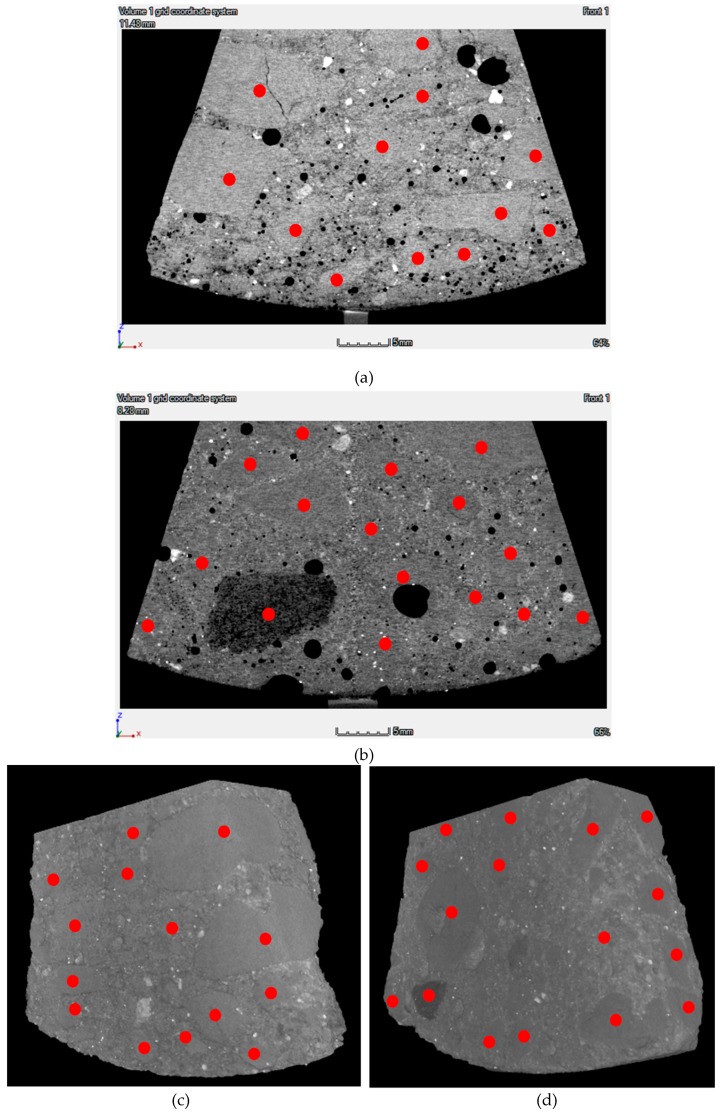
The 2D images for (**a**) C47 and (**b**) S42 and computed 3D images for (**c**) C47 and (**d**) S42. The red circles mark the visible aggregates.

The technique of X-ray microcomputed tomography (μCT) was applied to the concrete samples (“pizza slices”) to screen the interface transition zone between pipe and concrete. The primary goal was to determine quantitatively the thickness of the lubricating layer and the size and volume of the aggregates. Data from numerous X-ray radiographs was also processed to produce 3D images of the volumes under investigation. Various resolutions were applied to capture the distribution of particles in the lubricating layer and its transition to the plug region based on density differences between matrix and aggregates. Figure 7 depicts 2D and 3D images obtained for the reference mixtures, C47 and S42. The main challenge is to distinguish the coarse aggregates from the matrix, which contains cement paste and fine aggregates (due to the similar density of hydrated cementitious mortar and quartz aggregates).

2D images and computed 3D images specified most aggregates larger than 4 mm. Aggregates with sizes between 2 and 4 mm were detectable in some cases. However, the margins between the coarser aggregates and the surrounding fine mortar could not be assessed quantitatively. The authors also observed that particles smaller than 2 mm—sand, fine sand, unhydrated binder, and hydration products—were densely packed, and therefore it was difficult to distinguish the position of these particles. Hence, the methodology was found unsuitable for deriving the thickness of the lubricating layer containing particles finer than 1 mm.

## 4. Results and Discussion

### 4.1. Impact of Aggregates’ Volume-Fraction on Rheological Properties of Concrete and Constitutive Mortar

In this study, the authors considered the mortar as the suspending medium (liquid phase) and the aggregates as the suspended phase (solid phase) of the concrete. The measured Bingham parameters for the both mortar and concrete are summarized in Table 3.

**Figure 8:**
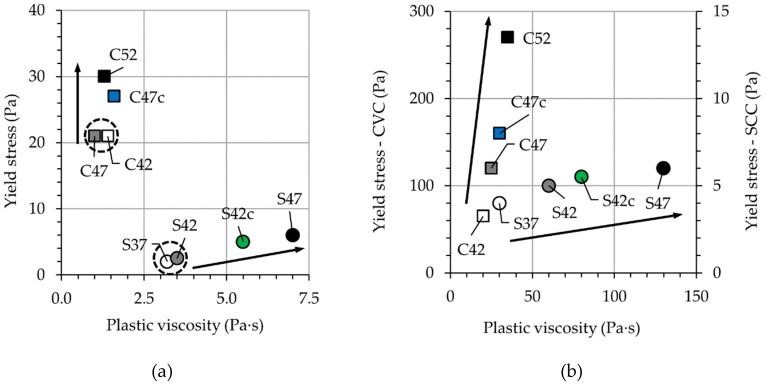
Yield stress and plastic viscosity for (**a**) mortar and (**b**) concrete. Arrows indicate the increase in reduced volume-fraction $\phi /\phi_{c}$.

For the sake of clarity, the rheological parameters are also represented using rheographs in Figure 8.

Based on the composition, the ratios among the solid constituents, the water-to-binder ratio (w/b), and the dosage of superplasticizer were kept constant for the mortars. C42 and S37 with volumetric aggregate content reduced by 5% showed similar rheological behavior to that of C47 and S42; see Figure 8a. However, this is not the case for mixtures C52 and S47 having 5% higher aggregate contents than the reference mixtures. This holds true also for C47c and S42c with crushed aggregates. For these four mixtures, both yield stress and plastic viscosity tend to increase; in CVC, the yield stress was significantly influenced while for SCC it was the plastic viscosity. Some increase in the rheological parameters of mortars could be observed with increasing aggregate content in concrete and replacement of round aggregates by crushed aggregates. These changes can be likely traced back to a more pronounced adsorption of the cement paste by coarse aggregates in those mixtures due to higher surface area of aggregates > 1 mm. Consequently, the sieved mortar contained less cement paste and water. The composition analysis of the sieved mortars confirmed this hypothesis. First, the authors determined the water content of the sieved mortar by drying. Then the actual and nominal water contents of the mortar were compared. The difference showed the amount of water adsorbed by aggregate surfaces together with fine solid particles. As a result, the additional 5% of aggregates of the mixtures C52 and S47 decreased the content of water in the corresponding mortars by 0.72% and 0.82%, respectively, in comparison to the reference mixtures. The rough surface of basalt aggregates in C47c and S42c also resulted in less water in the mortars: by 0.52% and 0.47%, respectively.

Investigation of the concrete mixtures shows very clear tendencies: an increase in the volume-fraction of aggregates or replacement of round aggregates with crushed ones results in significant growth of yield stress and plastic viscosity for both CVC and SCC. The arrows in Figure 8b indicate the increase in $\phi /\phi_{c}$ for each type of concrete. Moreover, the intensity of shear-thickening (c/μ) decreases with increasing volume-fraction of coarse aggregates in SCC mixtures. This can be explained by assuming the formation of (hydro-)cluster as the main mechanism for shear-thickening behavior of SCC. For compositions with larger aggregates, the coarse particles damage the clusters formed in the cement paste due to strong inertial forces acting on them and high shear forces occurring among them; thus, they reduce the intensity of shear-thickening [40]. By increasing the volume of the aggregates and reducing the volume of the mortar, firstly, there are fewer clusters to begin with, and secondly, there are more particles that can break down the clusters formed. In the case of S42c, replacing the round with crushed aggregates results in less intense shear-thickening as well. This can be traced to the higher shear rates developing among the aggregates of irregular shape.

### 4.2. Concrete Rheology during Pumping

**Figure 9:**
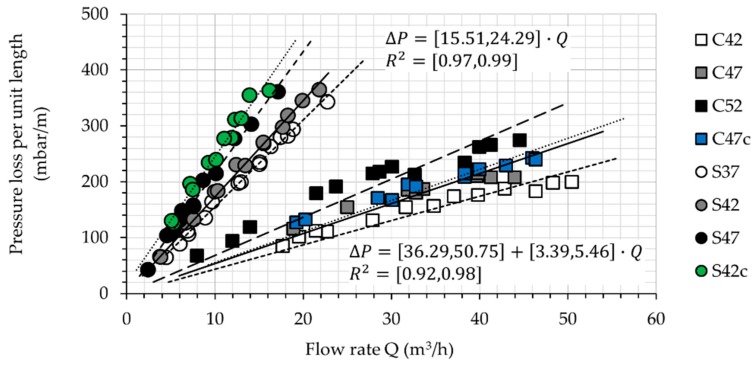
Required pressure for pumping one meter of concrete in a DN125 pipe with the desired discharge rate. The relation between pressure loss and discharge rate as well as R^2^ are given as range equations, showing minimum and maximum values.

During each stroke of the Sliper, only 0.5 m height of the material is being deformed (“pumped”) during the experiment. By dividing the observed pressure values by 0.5 m, the pressure loss per unit length in a DN125 pipe can be calculated and drawn at different discharge rates and for each composition. Figure 9 illustrates these data points and the linear correlation between ΔP/L and Q for all mixtures.

The thickness of the lubricating layer $e_{LL}$ was analytically calculated by fitting the ΔP/L−Q data points in Equation (6) similar to [41]. The rheological parameters of bulk and lubricating layer were set to the experimental values for concrete and mortar (Table 3), respectively. Since Equation (6) disregards the non-linear term c of the modified Bingham model for SCC mixtures, the rheological results were evaluated once more using linear regression, and the approximated Bingham parameters were used to determine the thickness of the lubricating layer analytically. For each ΔP/L−Q data point, one $e_{LL}$ value can be derived. The average of these values is reported as the thickness of the lubricating layer for each individual mixture in Table 4.

Note that the values listed in Table 4 are not the actual thickness of the lubricating layer during pumping. Rather, in combination with the measured rheological parameters of mortar, they can be used as a calibration parameter for predicting the pumping pressure in a full-scale pipeline. Both rheological parameters used in Equation (6) as well as in the flow equation derived by Kaplan et al. [17] are device-dependent. This means that for the same concrete, two different rheometers can deliver different sets of rheological parameters, and consequently, different ΔP/L−Q relationship. Ferraris et al. [42] showed that the rheological parameters can exhibit even ± 300% deviation depending on the testing device. The first question arises as to which rheometer can deliver the “real” material parameters. Which values can be used to derive a reliable relation between the pumping pressure and discharge rate? The answer is indeed none. Yet, to derive a reliable relation between pumping pressure and discharge rate for a full-scale pipeline based on the “uncertain” rheological parameters, one can use the analytical thickness of Sliper. A similar approach can be employed for a numerical simulation of a full-scale pipeline, see [37].

### 4.3. Experimentally Determined Thickness of Lubricating Layer

The actual thickness of the lubricating layer for each mixture can be obtained using the half-cylinder specimen tested in the Sliper; see Figure 5. Table 5 summarizes the average values and the corresponding standard deviation for each composition.

For each value reported in Table 5, at least 100 measurements were considered. The large standard deviation of the experimental thickness is mostly due to particles with a diameter of 1–2 mm that are positioned exactly at the pipe wall and taken into account when calculating average values and standard deviations. It is worth mentioning that the average thickness does not necessarily mark a region absent of particles coarser than one mm, but rather a region with a significantly reduced amount of such particles.

Considering the errors that might be introduced during casting of the samples as well as image analysis, the authors decided to analyze the qualitative pattern of the experimental values obtained. Reliable quantitative measurements are later required to establish a link between SIPM and the thickness and properties of the lubricating layer. According to the outcomes, the following conclusions can be drawn:

- With an increasing volume-fraction of coarse particles, the thickness of the lubricating layer decreases: the migration of coarse particles is hindered when the local volume-fraction of particles in the neighboring region reaches $0.8\;\phi_{c}$ [3,9]. With a larger ϕ, the equilibrium is reached faster and closer to the pipe wall; see Equation (8). Hence, a thinner lubricating layer develops ($e_{LL.S37}>e_{LL.S42}>e_{LL.S47}$ and $e_{LL.C42}>e_{LL.C47}>e_{LL.C52}$) [3].
- With an increasing rate of discharge, the thickness of the lubricating layer increases: at higher discharge rates, shear rate, and its changes with respect to radial axis ($\dot{\gamma }$ and $\partial \dot{\gamma }/\partial r$, as described by Equation (7)) increase, and so does the intensity of particle migration. Since higher discharge rates were measured for CVC than for SCC ($Q_{max,CVC}=3Q_{max,\;SCC}$), a thicker LL could be determined for CVC ($e_{LL,C42}>e_{LL,S42}$ and $e_{LL,C47}>e_{LL,S47}$), despite the fact of self-compacting concrete’s containing in general a higher volume-fraction of fine particles in comparison to conventional concrete ($\phi_{total,CVC}<\phi_{total,SCC}$); see Figure 9. The total content of fine particles, including binder and fine sand, in S42 and S47 was 4.59% and 4.29% higher than that in C42 and C47, respectively.

**Figure 10:**
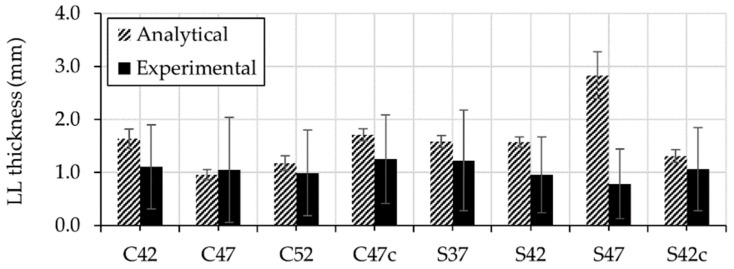
Comparison between experimentally and analytically determined thicknesses of the lubricating layer for all concretes under investigation.

Figure 10 illustrates a comparison between experimentally and analytically determined thicknesses of the lubricating layers.

Accordingly, the analytical values are in the scatter range of their experimental counterparts, except for the mixture S47. It is worth mentioning here that the analytical thickness is based on the rheological parameters measured using rheometers with relative measuring systems, coaxial cylinders. The analytical thickness was calculated under the following assumption: the rheological parameters of the concrete bulk and lubricating layer during pumping correspond to the rheological parameters of concrete and its constitutive mortar measured using relative rheometers. However, this assumption is erroneous under the consideration of SIPM. Instead, the rheological parameters of concrete bulk and lubricating layer vary in the radial direction depending on the local volume-fractions of particles. These changes influence the experimental thickness of the lubricating layer formed during pumping. Therefore, the analytical and experimental thickness of the lubricating layer might differ depending on the intensity of the SIPM. In the case of S47 with the highest volume-fraction of solid particles, the rheological parameters differed extensively inside and outside the pipeline. The reduced volume-fraction of solids ($\phi /\phi_{c})$ was close to the percolation threshold, and therefore, slight particle migration resulted in dramatic changes of the rheological properties; more information is to be found in Section 4.5. As a result, the analytical and experimental thickness vary significantly when compared to other mixtures.

It must be noted that the thickness of the lubricating layer also depends on our definition of this layer. If the lubricating layer is defined as the region consisting of fine mortar, its thickness depends on the intensity of SIPM. If the thickness of the lubricating layer is defined as the width in which the velocity increases significantly, studies indicate that the thickness is about 2 mm [6,43,44]. In this case, the composition of the lubricating layer within this 2 mm is not constant but varies based on the intensity of particle migration at different discharge rates. In both cases, a clear image for concrete pumping cannot be obtained without considering SIPM.

### 4.4. Particle Distribution in Radial Direction

**Figure 11:**
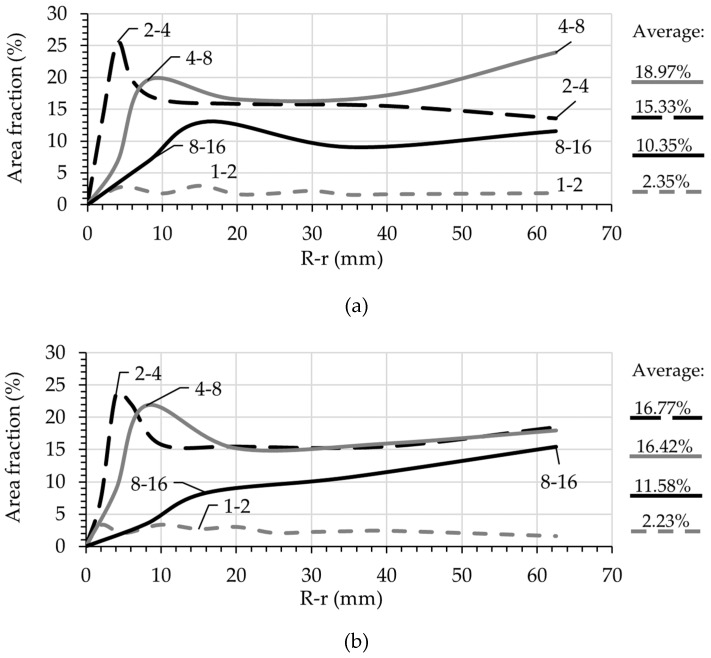
Surface area occupied by different groups of particles over the radial direction after pumping for (**a**) S47 and (**b**) C47. The overall average for each size is listed on the right side of the graph.

The surface area occupied by aggregates of a different size range was used for representing the particle distribution over the pipe radius. Figure 11 shows exemplarily these results of particle size distribution for the mixtures S47 and C47. The average volume-fraction was assumed to be equal to the average surface area fraction for each size listed on the right side of each graph. Note that both mixtures have the same particles size distribution.

Generally, if all the particles larger than 1 mm are traced, it is expected that the average area fraction for each group is equal to its volume-fraction in the mixture composition. However, due to the irregular shape of the particles, the tip of a larger particle can be erroneously classified as belonging to a smaller particle size range. The following remarks should be considered when using the present methodology:

- The number of traceable particles within the range 1–2 mm was often limited to one-fourth (1/4) of the expected values (expected: $\phi_{1-2}=0.0886$, measured values: 0.0235 for S47 and 0.0223 for C47). Therefore, the tracking of particles smaller than 2 mm in this method would not provide any additional information.
- The average surface area fractions for the particle groups 2–4 mm and 4–8 mm are larger than the expected values for both mixtures, expected: $\phi_{2-4}=0.0886$ and $\phi_{4-8}=0.1226$. On the contrary, for the particle group 8–16 mm, the average surface area is always less than expected. This is due to the irregular shape of the particles and their random orientation in the target cross-section.
- For particle size ranges 2–4 mm, 4–8 mm, and 8–16 mm a clear peak can be observed. It seems that the larger the particles are, the greater the distance of the peak to the pipe wall. Furthermore, the peaks are shifted from the maximum aggregate size for each group. This indicates that both wall-effect and SIPM influenced the radial displacement of the particles.

To increase the accuracy of this method, the particles of each group should have a different texture or color. The aggregates can be composed of quartz aggregates of 2–4 mm, basalt aggregates of 4–8 mm and granite aggregates of 8–16 mm. Alternatively, model concrete containing glass beads of different colors can be used to determine the particle distribution that is resulted from SIPM.

### 4.5. Evaluation of Surface Profile during Sliper Tests

The deformation and flow pattern of the top surfaces of the concrete mixtures were captured when tested in the Sliper. The average radial velocity of the coarse particles and the radius of the plug region were determined; cf. Table 6. Additionally, the analytical plug radii were also calculated using $R_{Plug}=2\tau_{0,B}/\Delta P$ and listed; more details can be found in Equation (6).

Since the reported radial velocities in Table 6 were determined at different pressure losses, the values were normalized according to the maximum pressure loss measured for S47.

(9) $$\upsilon_{r,norm.}=\upsilon_{r,avg}\cdot \left(\Delta P/\Delta P_{S47}\right)$$

**Figure 12:**
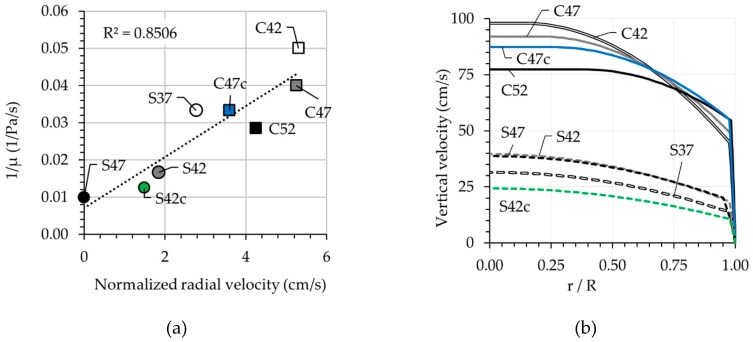
(**a**) Relation between normalized radial velocity at $\Delta P_{max}=22.996$ kPa/m according to the pressure sensor from Sliper and the inverse of bulk viscosity and (**b**) velocity profiles determined analytically.

In Newtonian fluids, the velocity profile and discharge rates can be calculated as:

(10) $$\upsilon_{z}\left(r\right)=\Delta PR^{2}/4\eta \;\left[1-{\left(r/R\right)}^{2}\right]$$

(11) $$Q=\int_{A}^{\;}\upsilon_{z}\;dA=\pi \Delta PR^{4}/8\eta$$

with $\upsilon_{z}\left(r\right)$ and Q as the vertical velocity at radius r (m/s) and total discharge rate (m^3^/s). ΔP, R, and η are pressure loss per unit length (Pa/m), the radius of the pipe (m), and the viscosity of the Newtonian fluid (Pa·s). Equation (10) shows that the focal width of the velocity parabola is directly proportional to the ratio between the pressure loss and apparent viscosity ΔP/η. To check whether this holds for non-Newtonian fluids as well, the inverse of plastic viscosity and the normalized radial velocities are compared in Figure 12a. The results show good correlation between these two parameters. The non-zero ordinate intercept is due to the formation of the plug during concrete flow.

The velocity profiles were calculated using Equation (6) for all mixtures and are shown in Figure 12b. Based on the graphs, for CVC mixtures, the analytical plug radii are between 0.13R and 0.37R under indicated ΔP. However, the experimental plug radii were zero. This is to be expected due to the smaller plug radii on the top surface when compared with sample inner region. For SCC mixtures, due to small yield stress values the analytical plug radii approach zero for all mixtures. This outcome agrees with the experimental radii for the mixtures S37, S42, and S42c.

In the case of S47, with a yield stress of 6 Pa and plastic viscosity of 130 Pa·s, the authors observed distinct plug flow with particle displacements of less than 5 mm during the drop. This mixture had the greatest amount of solid particulate with 83.86% by volume in total and 62% of particles larger than 0.06 mm, without binders. The maximum packing fraction of solids larger than 0.06 mm was determined experimentally as 79.74%, which results in initial $\phi /\phi_{c}=0.78$. According to [3], particle migration stops after a critical deformation of $\gamma_{c}$, when $\phi /\phi_{c}$ reaches 0.80. The critical deformation $\gamma_{c}$ is of order of $R_{Pipe}^{2}/10a^{2}\phi^{2}$, which in our case results in $\gamma_{c}$ of approximately 17 for an average particle size of 8 mm. The shear strain inside the sample can be simplified as

(12) $$\gamma_{sample}=H_{S}/d_{sheared}$$

where $H_{S}$ is the height of the sample equal 0.5 m (upper pipe) and $d_{sheared}$ is the sheared gap during each stroke, which corresponds to $R_{pipe}-R_{plug}$. Here, the plug radius is not constant: the moment the concrete enters the pipeline, $R_{plug}$ can be approximated as the analytical plug radius computed using the measured yield stress. Subsequently, the particles migrate toward the centerline, increasing the local volume-fraction and hence the local yield stress [12,13,14]. Any further strokes, pipe movements in Sliper, cause the additional migration of particles and increase of local yield stress and $R_{plug}$. At $t=T_{c}^{FIPM}$ the particle migration becomes steady, while local yield stress and $R_{plug}$ reach equilibrium. In the case of S47, the authors assumed an initial $R_{plug}$ of 0.04 cm; cf. Table 6. The shear strain inside the sample can be approximated to:

$$\gamma_{sample}=\frac{H_{S}}{d_{sheared}}≅\frac{0.5}{0.0625-0.0004}≅8$$

Comparison of $\gamma_{c}≅17$ and $\gamma_{sample\;}≅8$ indicates that with three strokes, the particle migration in S47 reaches a steady flow state. Therefore, the yield stress and plastic viscosity of the sample during the stroke specified in Table 6 were different from the experimental values mentioned in Table 3. Instead, they were product of the SIPM that happened inside the Sliper device during the first few strokes. S47 is an example of a modern concrete with a high volume-fraction of solid particles, whose rheological properties change significantly during pumping due to SIPM.

Until now most researchers have assumed that the variations in rheological parameters due to SIPM are not significant enough to influence the flow properties inside a pipeline. Thus, the assumption of two immiscible single-phase fluids—concrete bulk and the lubricating layer—with constant rheological parameters was sufficient to predict pumping behavior. However, the results observed in this discussion indicate that for mixtures such as S47 with a high volume-fraction of solid particles, the changes in the local volume-fraction of solids, and as a consequence, the changes in the local rheological properties are severe enough to alter the pipe-flow regime. Therefore, it is of utmost importance that the variations of local rheological parameters due to SIPM be considered in the pipe-flow equations.

## 5. Conclusions and Outlook

The aim of this paper was to capture the formation of the lubricating layer during the pumping of concrete and analyze it with regard to shear-induced particle migration (SIPM). Two approaches were suggested for capturing the lubricating layer thickness of concrete. Additionally, the rheological properties and pumping behavior of concrete mixtures were investigated with respect to the volume-fraction of aggregates. The experimental outputs were compared with the state-of-the-art in concrete pumping and the analytical flow formulations developed for concrete mixtures. Finally, the flow patterns captured on the surface of the “pumped” samples in the Sliper device were studied with respect to rheological properties and particle migration. The following conclusions can be drawn:

- The Bingham rheological parameters of concrete increase with increasing aggregates’ volume-fraction (ϕ). For CVC the changes are pronounced in the yield stress values in the first place, while in the case of SCC it is the plastic viscosity, which alters significantly.
- The thickness of the lubricating layer was measured on hardened concrete samples investigated after performing the Sliper tests. The thickness of the lubricating layer was not constant for the concrete compositions under investigation and varied under different discharge rates. The thickness of the lubricating layer increases with decreasing aggregate volume-fraction and with increasing discharge rate.
- The analytical thickness of the lubricating layer can be calculated using the rheological properties of concrete bulk and the lubricating layer and the output of Sliper. However, these values do not represent reality. The analytical thickness is suitable for eliminating the device-dependency of experimental rheological parameters for analytical or numerical modeling of the pumping process.
- Concrete plastic viscosity is the most influential parameter affecting the shape of the parabolic curve of velocity profile.
- In modern concretes with high volume-fractions of solid particles, the shear-induced particle migration can be severe enough to influence the local rheological properties of the pumping concrete and therefore, must be taken into consideration. In such cases, conventional analytical models using the rheological parameters obtained by means of concrete rheometers, are not sufficiently reliable to predict concrete pumping behavior.

Understanding and implementation of SIPM for a polydispersed suspension such as concrete with a continuous particle size distribution in analytical flow formulation requires further experimental investigation. In the next phase, it is planned to replace coarse aggregates by colored and spherical glass beads. This approach should increase the accuracy in investigating the influence of aggregates’ content and properties on particle distribution due to SIPM and the thickness of the resultant lubricating layer.

## Figures and Tables

**Table 1:** Compositions of concretes under investigation and their constitutive mortars as well as the concretes’ properties in the fresh and hardened states.

Materials		Density (kg/m^3^)	Dosage (kg) for 1 m^3^ of Concrete							
			C42	C47	C52	C47c	S37	S42	S47	S42c
**Constitutive mortar**	CEM III/A 42.5 N	2990	425	388	351	388	392	361	330	361
	Fly ash	2200	-	-	-	-	239	220	201	220
	Quartz powder	2680	57	52	47	52	52	48	44	48
	Quartz sand 0.06–0.2 *	2650	57	52	47	52	52	48	44	48
	Quartz sand 0–1 *	2650	456	416	376	416	420	387	354	387
	Water	1000	211	192	173	192	180	166	152	166
	Superplasticizer (SP)	1056	3.48	3.18	2.87	3.18	11.14	10.26	9.38	10.26
**Aggregates**	Quartz sand 1–2 *	2650	210	235	260	260	185	210	235	210
	Quartz sand 2–4 *	2650	210	235	260	260	185	210	235	210
	Quartz gravel 4–8 *	2650	290	325	360	360	255	290	325	290
	Quartz gravel 8–16 *	2650	403	451	500	-	355	403	451	-
	Basalt 8–11 * (crushed)	2900	-	-	-	197	-	-	-	176
	Basalt 11–16 * (crushed)	2900	-	-	-	300	-	-	-	268
w/b (-)			0.50	0.50	0.50	0.50	0.30	0.30	0.30	0.30
SP % bwob **			0.72	0.72	0.72	0.72	1.77	1.77	1.77	1.77
ϕ of agg. (> 1mm) (-)			0.42	0.47	0.52	0.47	0.37	0.42	0.47	0.42
$\phi /\phi_{c}$ (> 1mm) (-)			0.585	0.654	0.724	0.667	0.515	0.585	0.654	0.596
FT/SF for concrete ^†^ (mm)			640	545	450	530	720	680	600	650
FT for constitutive mortar ^‡^ (mm)			260	260	240	240	300	290	250	250
Air content (%)			1.7	2.7	1.3	1.6	0.7	0.8	0.4	1.0
Density (kg/m^3^)			2340	2320	2390	2370	2320	2360	2370	2410
Compressive strength at 28 d (MPa)			54.3 ± 1.3	50.8 ± 1.0	48.6 ± 1.1	53.7 ± 0.6	76.9 ± 1.6	78.6 ± 2.0	72.1 ± 0.7	81.1 ± 1.1

* The numbers indicate the range of the particle size in mm. ** Superplasticizer, percentage by the weight of binder. ^†^ Flow table spread (FT) for conventional vibrated concrete (CVC) based on DIN EN 12350-5 [29] or slump flow (SF) for self-compacting concrete (SCC) based on DIN EN 12350-8 [30]. ^‡^ Flow table spread (FT) for constitutive mortars based on DIN EN 459-2 [31], with 15 strokes for conventional mortars and without stroke for self-compacting mortars.

**Table 2:** Detailed information on the employed rheometers.

Device	Material	Geometry	Testing Profile	Transformation Equation	Torque Resolution	Angular Resolution
ConTec 5 viscometer *	Concrete	Serrated beater $R_{o}$ = 145 mm $R_{i}$ = 100 mm H = 120 mm	Hysteresis loop with rotational velocity range: 0.02 to 0.62 rps	Reiner-Riwlin [1,2]: $\begin{array}{c} T=G+H\cdot N\\ \tau_{0}=\frac{G}{4\pi h}\left(\frac{1}{R_{i}^{2}}-\frac{1}{R_{o}^{2}}\right)\frac{1}{\ln\left(R_{o}/R_{i}\right)}\\ \mu =\frac{H}{8\pi^{2}h}\left(\frac{1}{R_{i}^{2}}-\frac{1}{R_{o}^{2}}\right) \end{array}$	0.1 Nm	126 mrad
HAAKE MARS II rheometer **	Mortar	Hollow vane $R_{o}$ = 35 mm $R_{i}$ = 26 mm H = 50 mm	Hysteresis loop with rotational velocity range: 0.01 to 0.50 rps	Affine translation and calibration by reference materials [33]	0.1 nNm	12 nrad

* Produced by ConTec, located in Reykjavik, Iceland. ** Produced by Thermo Fisher Scientific, Karlsruhe, Germany.

**Table 3:** The Bingham rheological parameters for CVC and the modified Bingham (MB) rheological parameter for SCC mixtures.

	Parameters	Mixture							
		C42	C47	C52	C47c	S37	S42	S47	S42c
**Concretemortar**	Yield stress (Pa)	65	120	270	160	4.0	5.0	6.0	5.5
	Plastic viscosity (Pa·s)	20	25	35	30	30	60	130	80
	Non-linear term of MB (Pa·s^2^)	-	-	-	-	5.5	5.0	1.0	5.5
**Mortar**	Yield stress (Pa)	21	21	30	27	2.0	2.5	6.0	5.0
	Plastic viscosity (Pa·s)	1.4	1.0	1.3	1.6	3.2	3.5	7.0	5.5
	Non-linear term of MB (Pa·s^2^)	-	-	-	-	0.3	0.2	0.5	0.6

**Table 4:** Average analytical thickness of the lubricating layer.

Mixture	C42	C47	C52	C47c	S37	S42	S47	S42c
$e_{LL}\;$(mm)	1.63	0.96	1.17	1.71	1.58	1.57	2.83	1.31
Standard deviation (mm)	0.18	0.10	0.14	0.12	0.11	0.10	0.44	0.12

**Table 5:** Average experimentally determined thickness of the lubricating layer.

Mixture	C42	C47	C52	C47c	S37	S42	S47	S42c
$e_{LL}\;$(mm)	1.11	1.04	0.98	1.25	1.22	0.95	0.78	1.06
Standard deviation (mm)	0.79	0.99	0.81	0.84	0.95	0.71	0.66	0.79

**Table 6:** Average radial and vertical velocity of surface, and the plug radii for specified pressure loss.

Mixture	C42	C47	C52	C47c	S37	S42	S47	S42c
Pressure loss ΔP (mbar/m)	153.89	184.90	226.20	194.84	163.72	229.96	277.14	185.39
$\mathrm{Average}\;\mathrm{vertical}\;\mathrm{velocity}\;\upsilon_{z,avg}$ (cm/s)	71.69	72.18	68.13	72.18	21.93	28.14	27.73	17.00
$\mathrm{Average}\;\mathrm{radial}\;\mathrm{velocity}\;\upsilon_{r,avg}\;$(cm/s)	7.93	6.53	4.31	4.24	3.90	1.85	0.00	1.85
Exp. $R_{plug}\;$(cm)	0	0	0	0	0	0	5.53	0
Analyt. $R_{plug}\;$(cm)	0.84	1.30	2.39	1.64	0.04	0.04	0.04	0.04
